# A Practical Work on the Diseases of the Eye, and Their Treatment, Medically, Topically, and by Operation

**Published:** 1840

**Authors:** 


### A Practical Work on the Diseases of the Eye, and their Treatment, Medically, Topically, and by Operation. By *Frederick Tyrrell*, Senior Surgeon to the Royal London Ophthalmic Hospital, Surgeon to St. Thomas's Hospital, Professor of Anatomy and Surgery at the Royal College of Surgeons in London, &c. London: John Churchill. 2 Vols. 8vo. 1840.

Mr. Tyrrell's opportunities of observation at the London Ophthalmic Hospital have necessarily been great, and no less great than valuable. The work before us affords sufficient evidence that he has not neglected them.

There are two sorts of works on the science of medicine—the one offers the results of the experience of the author, with little parade of learning and no critical examination, at least no elaborate criticism of the views of others—the second kind of work is stuffed with references, swollen with quotations, and mottled with the various notions of various men. Both works are useful, both necessary, and either would be incomplete without the other. But if the first kind of book is well executed, and if the experience it conveys has been considerable, we cannot be surprised at the preference which practical men are apt to shew towards it.

Every body is aware that there is a class of bookworms who affect to look down upon those practical men. We know not what right they have to do so. A commentator and a man of indices is constructed without difficulty. Plodding industry, with a very moderate amount of sense, are the raw materials, and even the manufactured article is made of little more. But practical talent in any art, and more especially in one like medicine, a calculation as it is, of chances, implies some mental requisites, we might almost say accomplishments, that none but fools despise. Quickness of perception, rapidity of inference, and no trifling decision, are the natural gifts on which must be grafted the acquired knowledge of the art. Those who think lightly of practical talent have it not, or are jealous and insincere in their contempt of it.

Let us not be understood to sneer at learning. Those who *know* us will not tax us with that. But to set up a mere acquaintance with other men's opinions over the power of forming one's own, is to give to one species of knowledge a superiority, that, neither in the eye of reason nor of usefulness, it is entitled to.

Mr. Tyrrell's volumes are of the plain matter of fact description that we have alluded to. They convey the experience he has acquired in a simple, intelligible form, with few graces of style, and no effort at learning. The reader will become acquainted with *his* practice, learn *his* opinions, and approve or disapprove as he chooses. The reader will *not* learn the opinions and the practice of every previous writer, and, if he wants this, he must seek for it elsewhere.

Mr. Tyrrell informs us that, in treating of affections of the eye, he has endeavoured, for the most part, to state the most prominent and least variable symptoms; such as are, in fact, most characteristic of each particular disease; he has purposely avoided entering into a detail of the more minute and more variable signs, which tend so much to confuse in description, and which, really, are of little value in practice.

Perhaps the work before us wears too much of the garb of the lecture-room, and displays somewhat a magisterial tone in matters that are familiar to all well-informed men. Mr. Tyrrell, for example remarks:—

"The means of obtaining a correct diagnosis, which should determine and guide the plan of treatment, in any particular local disease, should, in my opinion, comprehend, not merely the investigation of the local signs or symptoms; but also include an enquiry into the condition of the system generally, and of the principle and important organs, and their functions; by derangement of which, local disease is so frequently modified and maintained." xxvi.

Which is all very true, but no more true than trite. His observations on plethora and debility partake of the same character—a kind of assumption of ignorance on the part of the auditor, which, though necessary in the wards and in the class-room, is not so beseeming in a book addressed to the profession. We pass over all this sort of thing, recommending it, however, to the junior student, on whom Mr. Tyrrell's remarks cannot fail to make an impression, and we proceed to a chapter of *Preliminary Remarks*, which contains many useful hints.

1. *Mode of Examining the Eye.*—There can be no doubt this is often done clumsily enough, and injection, irritation, and additional inflammation of the eye induced by it. In a notice which we gave of Mr. Morgan's book, we quoted his method of examining the eye; and Mr. Tyrrell's is not very dissimilar. Perhaps it would be as well to mention it.

The patient, says Mr. Tyrrell, should not be exposed to too great a body of light; nor to a bright or direct light; but only to such a degree of it, as may be sufficient to afford a correct view of the organ; and the patient should, if possible, be placed, so as to receive the light, obliquely, upon the face. If an adult, either the reclining or recumbent posture is best—as enabling the medical practitioner to look well beneath the superior palpebra. The patient should be directed to cover one eye with a handkerchief, or with the hand, whilst the other undergoes an examination, which should be conducted in the following manner: —the point of the fore-finger, of one hand, should be placed a little below the centre of the inferior palpebra, about the margin of the orbit; so that by pressing the integument downwards, and, at the same time, backwards, or towards the maxillary bone, sufficient stress can be made on the skin, to cause its depression of the lid, and slight eversion; simultaneously, the extremity of the thumb, of the opposite hand, should be placed near the centre of the superior palpebra, a little above the upper margin of the tarsus, between it and the eyebrow; so that little or no pressure be made upon the globe itself, but so as to enable the examiner to raise the integument of the lid towards the brow, against which he may make firm pressure; and thus, by acting upon the integument, he may raise the palpebra to a sufficient extent, to obtain a satisfactory view of the eye, without any violence to the globe: the finger and thumb of the two hands should act together, to depress the lower, and elevate the upper palpebra at the same time; and during the separation of the lids, the patient should be directed to look downwards, inwards, and outwards, in succession; by which the whole cornea can be brought under observation.

Another plan is required with a child. It should be placed upon its back, across the nurse's knees, with the head projecting on one side, towards the examiner, who should be seated, in a high chair, at the side of the nurse, so that he can receive the child's head between his knees; he can thus command the child's head, whilst the nurse commands the body and arms.

2. *General Treatment.*—Mr. Tyrrell devotes some pages to this. We need not follow him in detail, but merely allude to a point or two.

*General Bleeding* he looks on as unnecessary and improper, unless the pulse evinces a degree of *resistance or incompressibility*, besides an unusual degree of fulness, or quickness; except when there is cerebral mischief or disease, when the pulse is usually slow and labored. He thinks that, no more blood should be taken than just suffices to relieve the state of tension, or to diminish the force of the circulation.

"In most instances, faintness does not occur when sufficient blood has been taken away, to produce the requisite change in the character of the pulse; and frequently the patient does not experience much immediate relief as regards the local disease; but after a short time, the beneficial influence of the treatment becomes apparent, in a decided mitigation of the severe local symptoms; and the cure can be generally completed in a shorter period, than when the loss of blood has been so great as to cause a state of depression: for, although the effects of bleeding to such an extent, as to occasion prostration of strength, usually produces a more decided and rapid relief of the urgent local symptoms, yet the morbid action which remains, commonly proves obstinate or difficult of cure, in proportion to the extent of the general debility which ensues from abstraction of blood."

Mr. Tyrrell thinks it a great mistake to suppose that it is requisite to draw blood in large quantities. It will be observed that these opinions clash directly with those of Mr. Lawrence and others of that school. We believe, however, and have long done so, that the moderate practice is the best, and we cannot say that we coincide with those who bleed à la Sangrado in inflammatory eye diseases.

From amidst some observations on the use of mercury we extract the following:—

"Very many surgeons imagine that mercury acts most beneficially whilst the patient is kept upon a very abstemious diet, and that generous living is incompatible with the use of this remedy: I am, however, perfectly satisfied that it acts more certainly and beneficially when a tolerable degree of power is maintained; and that there are very few cases in which mercury cannot be taken, provided that the patient be properly supported during the administration of the remedy: I consider it a general rule, that whenever it is necessary to give mercury for the continuance of a few weeks, that it is essential to promote and maintain power at the same time."

Our readers are aware that this is a point on which we have always insisted. If persons are to take a lengthened course of mercury great hazard is inflicted on them by keeping them low. On consideration, can it be deemed necessary? For protracted courses are not given for affections of an acute inflammatory character, and very chronic complaints are seldom benefitted by what greatly reduces the bodily powers. In primary and secondary syphilis, it is a fatal error to keep a patient very low. If his constitution is naturally weak, that weakness is augmented, perhaps seriously, by the debilitating influence of mercury and a low regimen. But if the disease is acute and of inflammatory character, then, of course, the action of mercury is assisted by antiphlogistic measures. Such a course of mercury, however, is *not* a long one.

3. *On the Application of Local Remedies.*

*a. Fomentations* should be of moderate heat (we suppose the best rule is to consult the patient's own feelings and to watch the effect). Mr. T. thinks that the best means of using the fomentation, is by soaking a piece of fine linen or flannel in the heated liquor, and then placing it in contact with the surfaces of the palpebræ, which should be closed as when asleep. Five or six minutes of time are quite sufficient for the continuance of the fomentation at once; and the part should be well dried, when the application is laid aside. Mr. Tyrrell objects to sponge because it may be gritty. It is easy, however, to tell that, and well to use it if it is *not* gritty. Steaming the eye is even preferable to fomenting it. If a large funnel be inverted over a jug containing the heated fluid, the steam which escapes from the narrow end of the tube, can be received against the palpebra, at a distance agreeable to the patient.

*Lotions.*—" I generally use a lotion cold or tepid, as may be grateful to the sensation of the patient; and, most frequently, find that the tepid preparation is selected. If a small cup or glass, containing a portion of the lotion, be placed in a larger vessel, partly filled with hot water, the lotion acquires the warmth of new milk, (which is sufficient,) in a few seconds. Either warm or cold, the lotion should be applied with a piece of soft linen, to the surfaces of the eyelids, which should be gently closed; when sufficient of the fluid will penetrate the palpebral aperture, to effect the desired purpose: I object to the use of lotions with the conjunctiva exposed, either when they are used by eye-glass, syringe, or other method: I have seen much mischief from their being thus employed. If a case require the free contact of a stimulant or astringent to the conjunctiva, I much prefer the application of a drop or two, through the palpebral opening. I am of opinion, further, that the application of a lotion should not be continued more than one or two minutes at a time, and never kept to the part for hours together, as I know they frequently are: too much moisture, especially with cold, is apt to induce affection of the fibrous structures.

Poultices are objectionable for the same reasons." xlix.

However, we have seen light poultices useful, and we doubt whether medicated lotions act very efficiently *as such,* when merely applied in the shape of wetted linen.

*Dry Warmth.*—Small square or rounded bags of linen or flannel, about three, or three and a half inches in diameter, should be filled about two-thirds full with bran, or camomile flowers; and with either of these, some narcotic, camphor, or other matter, as thought proper, can be mixed; when used, they should be put upon a hot plate, or a warming pan, and when sufficiently heated, one should be placed so as to cover the eyelids, brow, &c.; and retained by a gauze handkerchief or ribbon: the application may be continued as long as the patient pleases, (provided the bags be not made very hot,) as it is not likely to produce any mischief. It affords great relief in some cases.

Speaking of *ointments* and *oil*, Mr. T. objects to the latter, when medicated, as more likely to penetrate in quantity, between the palpebræ, to the surface of the eye, than an ointment, and this is not the intention of the application.

*Leeches*, says Mr. T. should be applied to the outer surfaces of the eyelids, or upon the cheek, a little below the inferior palpebra; they have very little effect in relieving the vessels of the conjunctiva, when placed upon the temple. We are not quite sure of this. We have seen leeches relieve conjunctival inflammation more when placed upon the temple, than when applied to the cheek. It has seemed to us that leeches often act as *derivative* agents rather than as direct emptiers of the inflamed vessels. And if this be the case, it is not necessary that they be applied on the course of those vessels. If a case is at all troublesome, we generally apply leeches below the eye *and* on the temple. By this means we get both derivation *and* evacuation of the turgid vessels.

Mr. Tyrrell has been compelled to abandon the application of leeches to the conjunctiva itself, as it more frequently produces mischief than good, by creating irregularity of the surface of the membrane, which gives rise to irritation as an extraneous body would. And for a similar reason scarification of the conjunctiva is not applicable to ordinary cases.

In cases requiring local abstraction of blood, when leeches cannot be procured, or when their use is forbid, the angular vein may sometimes be opened, with much advantage, but only when it is well developed; otherwise, the cupping glass should be applied to the temple, or behind the ear, a little below the mastoid process.

*Counter-irritants* are best applied behind the ear. Mr. T. recommends as a slight counter-irritant:—

℞. Liquoris ammoniæ fortiss. ʒij.

Olei amygdalæ ʒss.

Adipis recentis ʒvss. Misce.

It should be put into a wide-mouthed bottle, and well secured; or the ammonia soon escapes. When employed a small portion should be smeared over the forehead, above the eyebrows, by means of an ivory paper knife; and it should be allowed to remain on, till it produces a smarting or heat; (from half a minute to a minute;) it should then be washed off, by sponge and water. It may be repeated daily.

Mr. T. prefers repeated to open blisters. For children he frequently directs a few cotton threads to be smeared with some of the blistering ointment, and to be placed behind the ear during the night.

Mr. Tyrrell is afraid that when *issues* or *setons* are discontinued, the disorder is apt to return. When he does make an issue it is with beads in the arm.

He recommends, in conclusion, that "in all inflammatory and congestive diseases of the eye, the patient should not be confined to bed, or allowed to be much in the recumbent posture, if at all: for even when in bed, the shoulders and head should be raised and well supported. The relief obtained by position in the acute diseases is very great, and it is, frequently, taken advantage of by the patient, when neglected by the medical man; for I have often heard the sufferer observe that he had been sitting up in bed the greater part of the night, and that he had been easy, or his pain had been very much lessened, whilst he maintained such a position; but that his symptoms became aggravated, as soon as he had resumed the recumbent posture."

We fancy that this proscription of the recumbent posture is a little harsh. In severe inflammatory diseases of the eye the patient not unfrequently prefers bed, for in it there is an equality of temperature, freedom from exertion, and quiescence of the circulation not so satisfactorily obtained out of it. Much we suppose must depend on the patient's wishes and his feelings.

Mr. Tyrrell passes to the conjunctiva, the anatomy of which he describes, and then takes up its morbid conditions. He admits the following varieties of ophthalmia:—simple ophthalmia—pustular ophthalmia—catarrhal ophthalmia—purulent ophthalmia—chronic ophthalmia, not as a result of acute disease, but originating in chronic form—strumous or scrofulous ophthalmia—and exanthematous ophthalmia—the two last are modifications of most of the above varieties.

### SIMPLE ACUTE OPHTHALMIA.

There is nothing in the account of this to detain us, indeed the only point to which we would refer is the necessity for strict investigation of the constitutional state in the management of the disorder. Such a necessity is so obvious that, at the present day, one would suppose that it never is neglected. Yet, however obvious, it certainly may be, and occasionally is, permitted to escape attention, as several cases detailed by Mr. Tyrrell prove.

*Case.*—" A young lady, about eight years of age, of delicate form, was lately brought to me by her medical attendant, in consequence of her having ophthalmia, not severe, but still productive of much suffering, and sufficient to prevent her from using the eyes even for purposes of amusement; the affection had yielded in a degree to the use of leeches, purgatives, and a careful diet, but the relief had only been temporary. The obstinacy of the case under ordinary treatment, and a healthy condition of alimentary canal, induced the medical man to seek my opinion. I found that the only important deviations from her healthy condition was that her rest was disturbed, and that she was unusually hot at night, but this was attributed to the local disease, as, at the same time, she complained of increased pain or uneasiness in the eye. I was, however, satisfied that the local disease and rest were influenced by the defective cutaneous action, and I prescribed small doses of mercury with chalk and the compound powder of antimony at night, with an occasional mild aperient, a tolerably good diet, &c.; in a few days the ophthalmia was gone, her rest had become quiet and undisturbed, but at the same time the nocturnal heat and dryness of skin subsided, and the usual healthy moisture appeared."

The surgeon should carefully ascertain what is wrong, and endeavour to set that right. Yet, so far as we have seen, cases where some little particular symptom calls for some particular remedy, are not the rule, and the general treatment of an inflammatory disorder is generally the right.

Simple ophthalmia, observes our author, frequently exists with a condition of general power below the healthy or ordinary standard, and the local disease will not yield whilst this state of feebleness continues, but is so much dependent upon it, that the ophthalmia frequently subsides, as soon as general power is restored.

Very many cases of this kind result from over depletion in treating the common acute disease; others are consequent on exhaustion from febrile disease; indeed, any circumstance which reduces the constitutional vigor much below the healthy standard, will operate in maintaining this condition of conjunctival inflammation.

Mr. Tyrrell thinks that though a *low* this is rather an acute than a chronic affection, though it presents some peculiar characters:—The conjunctiva, for instance, exhibits numerous vessels, filled with red blood, of a dark or purplish hue; these vessels are very tortuous in their courses, and occupy the same positions as the principal vessels in the more acute form of ophthalmia, but they are less numerous, as well as more tortuous; the palpebral division of the membrane is also usually more red than natural; the organ is generally irritable, and the secretion abundant; there is however less pain, less heat, and less augmentation of suffering from the recumbent posture, than in common acute cases; but the aspect of the patient, the paleness and look of depression, with coldness of extremities, and feeble, but often rapid pulse, the loss of muscular power, restlessness, and sometimes inordinate cutaneous action, evince the deficiency of power.

We would observe that few well-informed surgeons persevere in depletion, when the colour of the conjunctival vessels grows dark, and an ulcer with little injection and no disposition to deposition is on the cornea, while the general state is one of lowness. General support, or, at least, sustenance, with mild local stimulants, we believe to be the common practice.

Mr. Tyrrell acknowledges that the most troublesome cases of ophthalmia that he has witnessed have been connected with some obscure peculiarity of system. The local disease has, in these instances, been principally confined to the ocular division of the membrane, and has, in most of the cases, extended to the cornea, producing slight superficial ulceration; further, the attacks of pain have been more severe than in the ordinary disease, and the organ more irritable; considerable intolerance of light has also existed, especially during the attacks of pain, which have had a neuralgic character.

The general peculiarity has consisted in an extreme feebleness of circulation, the pulse being quick, undefined, and so weak as to stop from very slight pressure; pallor, coldness, and loss of muscular power have afforded further proof of the low condition of vascular action.

The patients who have suffered from this form of disease, have all experienced intervals of relief from suffering and irritability of the eye, though it has still remained red and weak, and on careful observation, such intervals of relief have been often found to arise, from some atmospheric change, which has been favorable to the health of the individual.

The treatment has been principally directed to the promotion and maintenance of general power, by diet, exercise, clothing, and medicine. A case which Mr. T. relates is sufficiently illustrative of the affection.

*Case.*—"It was that of a gentleman, about forty years of age, who was engaged in one of the principal banking-houses of the metropolis; he had suffered for above three years, before the period of his consulting me, and had been under the care of most of the medical men of London who had directed their attention to ophthalmic diseases, but he had not obtained more than an occasional mitigation of the disease. After going through the history of the treatment he had undergone, I was much puzzled what course to pursue; for he had tried almost all varieties of general and local remedies. I could not detect any important functional derangement, but only a want of general power in the action of the heart and arteries; he expressed a feeling of weakness, but attributed it to the medical discipline he had undergone. The ophthalmia was not very severe, it affected both eyes, and was attended with much irritability of the organs; but it did not altogether incapacitate him from attending to his duties in the banking-house, though it produced a good deal of suffering and distress, and compelled him occasionally to give up business for some days together. The disease was much influenced by sudden change of weather, from dry to wet. I directed my treatment principally to improve and maintain the state of the circulation by attention to the secretions, a good and generous diet, and warm clothing, with the use of tonic medicine, which I varied the form of frequently, making a change whenever the form in use became offensive to the stomach, or failed in producing the desired effect. Yet with every care and attention I could not effect a cure; though I succeeded in getting rid of much of the irritability, and kept off any serious relapse, so that he could attend to his business with comparative comfort. After he had been under my care for several months, he, by my advice, consulted my colleague, Dr. Farre, who strongly urged a change of climate for a few months, as he considered that it afforded a prospect of a more decided and beneficial change in constitutional vigor; and, as I warmly supported the opinion of Dr. Farre, the patient made arrangements for an absence from business for three months, (the summer being at the time a little advanced,) and soon left England for the South of France; before, however, he arrived there, he experienced a beneficial change, both in the local complaint, and in general health or strength; and after a few weeks' residence in the South of France, he perfectly recovered, and much enjoyed the remainder of his holiday in wandering about; he returned to England at the end of the three months, in excellent health, and almost without trace of the ophthalmic disease. Many years have since elapsed, but I believe that he has not had any return of the disease of the conjunctiva."

Mr. Tyrrell relates several other cases, which however present no features essentially different from the preceding. In the majority of such cases, what is wanted is a steady perseverance in treatment, calculated to improve and maintain a proper degree of general power. He has found it necessary, occasionally, to change the form of tonic, and has obtained the greatest benefit from those of metallic quality, as the sulphates of iron, zinc, and copper, in very small doses; also from iodine and hydriodate of potash combined with sarsaparilla or some mild bitter, and the preparations of bark, quinine, &c.

### PUSTULAR OPHTHALMIA.

Mr. Tyrrell recommends, in the treatment of this affection, very simple measures:—a moderate diminution of diet; a mild purgative, the application of a weak astringent lotion of acetate of lead, or of alum, with the use of a mild ointment to prevent adherence of the eyelashes; and rest of the organs. In acute cases, a more active plan is required, as local bleeding by leeches, greater abstinence, and medicine to act on the principal secretions more freely; but the general power must be watched, and not allowed to get much below par, which it is apt to do. Mr. Tyrrell observes:—

"I have frequently seen this affection treated at an early period, by the application of a solution of nitrate of silver, of one, two, three, or four grains to an ounce of distilled water, and, in many instances, with marked good effect, the cure being rather more rapid than by the plan of treatment which I have described;—I have also more frequently seen this stimulating remedy fail, when the cases have been afterwards more protracted and obstinate.

It is, in my opinion, a very uncertain remedy, and as the disease is usually so easily and quickly relieved by the more simple and less severe plan, I cannot recommend the use of the nitrate of silver." 41.

We must say, that so far as we have seen, the gentle mode of treatment has answered best. At the same time we think it very frequently happens that, after a time, some mild stimulating lotion is serviceable. The sulphate of zinc has seemed to answer as well as any.

### CATARRHAL OPHTHALMIA.

For this Mr. Tyrrell recommends the plan of treatment advised for the pustular disease; but in the former case, the general remedies may be more active in the commencement; and locally, the solutions of alum or acetate of lead, besides being employed rather stronger, should be warmed before applied. Mr. T. usually prescribes poppy decoction with alum, (one pint and one drachm), as a lotion to be used tepid, every two or three hours.

Local bleeding is more frequently required in the catarrhal affection.

As the severity of the local disease subsides, the local applications should be gradually increased in strength, and a weak form of the citron ointment should be substituted for the simple unguent; this change tends very much to secure the patient from a chronic stage of disease. Mr. T. directs the palpebræ to be carefully dried with soft linen after the use of the warm lotion, for he thinks that the long-continued application of cold and moisture excites inflammation in the sclerotica.

Sometimes the acute stage proves obstinate, and Mr. T. has recommended change of air with good effect. He objects to the use of strong stimulating applications to the eyes. And we must confess that, after all that has been said in favour of them, we are very sceptical of their propriety.

### Purulent Ophthalmia.

Mr. T. describes, first, the Purulent Ophthalmia in the adult, then the disease as it occurs in the infant.

*Purulent Ophthalmia in the Adult.*—Mr. Tyrrell offers a very good description of the mode in which the cornea is destroyed in this disease.

"The principal supply of blood to this texture, is through the vessels of its conjunctival covering, as may be distinctly seen in some morbid conditions of these structures *(see corneitis, and vascular cornea, from granulated lid, or strumous ophthalmia)*, for, the larger vessels ramify in the conjunctival layer, and send minute branches to the texture of the cornea. The occurrence of chemosis, or elevation of the conjunctiva covering the sclerotic, around the margin of the cornea, by rapid deposition in the sub-conjunctival cellular membrane, first impedes, and, then, altogether interrupts the circulation in the corneal portion of the membrane; and this arises from the pressure, at the margin of the cornea, by the deposition, and from the tension and displacement of the membrane; for the attachment of the conjunctiva, over the junction of the cornea and sclerotic, is so firm, that it undergoes little or no change, whilst rapid and extensive alteration takes place in the membrane around—but its organization is so delicate, that the circulation is easily interrupted, by the combined effects of pressure and tension.

The cornea does not mortify from an extension of inflammatory action to it, but solely from interruption to its supply of nutritious fluid. Mortification of the cornea commences in various parts of the structure; most frequently near the margin, sometimes near the centre, or between the centre and circumference; in the first position, if the disease be arrested, the slough is usually of a crescentic figure, following the margin of the cornea; in the other positions, under similar circumstances, the slough is generally round or oval." 55.

Mr. Tyrrell, like the majority of surgeons, believes the disease contagious. He has seen many instances of gonorrhœal ophthalmia in the female. It is modified by the mode of origin—by the age and constitutional vigour of the patient—and by the co-existence of fever or other severe constitutional disturbance. Thus, he says, when the local affection is idiopathic, or induced by violence, or results from the contact of matter, and the patient is free from constitutional disorder,—the specific form, or that from application of morbid secretion to the conjunctiva, is very severe, and proceeds to the destructive termination much more quickly than the others. Most frequently under these circumstances one eye only is affected at first, and, though the second rarely escapes altogether, yet many days often intervene between the development of the complaint in the two.

If it arises from exposure to cold and damp, it is often attended with acute febrile symptoms, of catarrhal character, and, then, under the excited condition of the vascular system, the local disease proves severe and rapid in progress, and it usually attacks both eyes. Further, when it occurs with any of the acute febrile diseases, as small-pox, measles, scarlatina, &c. it is generally formidable and destructive.

*The Treatment* of the disease is considered by Mr. Tyrrell in reference to three stages of the complaint.

1. That without chemosis, or swollen palpebræ.
2. That with chemosis, and swollen palpebræ, but a clear cornea.
3. That in which mortification of the cornea has taken place.

The symptoms in the first stage might, without great circumspection, attract little attention. The surgeon should ascertain if the disease is attended with gonorrhœa, and should mark the general tone of the patient. If the circulation is vigorous, it should be moderated by venesection, and the effect should be kept up by abstinence, purgatives, and quiet. Great benefit may be obtained from two medicines—first, tartar emetic or ipecacuanha, in small doses, frequently repeated, to excite and keep up a condition of nausea, but not to produce vomiting, which would be injurious, as promoting ocular congestion. Second, mercurials, as tending to check deposit of fibrin, should the local disease advance. Persons of feeble power, of advanced age, of marked scrofulous diathesis, or females in an advanced stage of pregnancy, should not be submitted to loss of blood generally, but, be treated by abstinence, purgatives, nauseating medicines, and mercurials. Care must be taken, in all cases, not to let the general strength sink too low.

The head should be kept considerably elevated above the chest; such position will be found materially to mitigate suffering; leeches should be applied freely on the surfaces of the palpebræ and cheek, or, in case their use is forbidden, the angular vein should be opened, or a cupping-glass should be applied to the temple, or behind the ear; the local bleeding should be repeated on the recurrence of pain, or if the vessels become turgid, and of bright color. Warmth and moisture are serviceable for relaxing the vessels and membrane, and favoring a free discharge of pus; and may, at first, be repeatedly used, for short periods —as a minute or two: but so soon as the violence of the local symptoms has subsided, and the conjunctiva has lost some of the brilliancy and intensity of its colour, astringents should be employed;—alum is the best, which may be added to poppy decoction; at first, half a drachm to the pint, and this should be gradually increased in strength, up to six grains to the ounce, until the tumefaction of the membrane subsides; but it should be omitted on any recurrence of acute symptoms: throughout the treatment some mild ointment, or fresh lard, should be applied to the cilia and canthi, after cleansing the organ, or after using the fomentation or lotion. After the local abstraction of blood free blistering to the neck, or behind the ear, should be resorted to.

*Chemosis.*—The deposit which occurs in the sub-conjunctival cellular tissue, may be either fibrinous or serous: the former takes place in the most acute cases, and, by its firmness and the rapidity of its effusion, soon obstructs the corneal circulation; the latter, affording less resistance, and being thrown out with less rapidity, affects the supply of the cornea more slowly.

The disease in the second stage, with chemosis and swollen palpebræ, but a clear cornea, is hazardous, in proportion to the extent and character of the former. So long as the chemosis is incomplete, or does not embrace the whole circumference of the cornea, the risk to the cornea is not great; but, when the chemosis is complete, the life of the cornea is in momentary jeopardy; especially if the chemosis result from fibrinous deposit—the danger being less when the cellular tissue is distended with serum. The chemosis usually commences at the lower part of the surface of the globe, and encroaches on the temporal and nasal sides of the cornea; and lastly extends above it. In examining the affected organ, therefore, the attention should be directed to this spot.

When the chemosis is incomplete, Mr. T. recommends the means already mentioned. They do not fail if energetically used. Mr. T. is averse to the ultra depletory treatment recommended and practised by some surgeons. Nor does he like the strong stimuli or astringents patronised by others.

When the chemosis is complete the treatment is too often unsuccessful. "I have tried locally," says Mr. Tyrrell, "strong solution of nitrate of silver, and the undiluted solution of the diacetate of lead; but I have never seen the slightest advantage gained, by such remedies, in the state of the disease, in which complete chemosis existed: in the early stage, I grant, that powerful astringents are sometimes of much service, as they are in the catarrhal affection; but I consider their employment to be more frequently prejudicial than beneficial, adding, as it were, fuel to the fire, and do not, therefore, recommend their use, because the first stage of this disease is always curable by simple means."

Mr. Tyrrell advises scarifications of the conjunctiva. We lately noticed his views upon this matter at large. Scarifications relieve the tension of the membrane, and permit the cornea to receive its due vascular supply.

"I was aware that incision, and excision, of parts of the conjunctiva, had been suggested and effected, in the condition of chemosis; and, that the result of such treatment had not been very satisfactory; this want of success, however, appeared to me as a consequence of the mal-application of the principle, and not from error in the principle itself; for the incisions in the membrane, and excisions of portions of it, had been generally made in a direction corresponding to the margin of the cornea, and frequently extended completely around it, but at a short distance from it; thus the vessels passing to the corneal portion of the conjunctiva, must have been in great part, if not in toto, divided, and the supply of the corneal portion and cornea cut off, or nearly so;—the operation tended, therefore, rather to augment, than diminish the mischief, it was meant to obviate: this error arose from ignorance of or inattention to the anatomy of the organization of the part.

An attempt to relieve severe phlegmonous inflammation of the arm, by a circular incision through the integuments and cellular membrane, as in amputating, would, in my opinion, be just as likely to succeed, as the circular division of the conjunctiva, in the ophthalmic disease, now under consideration.

In the plan I proposed, the incisions were to be made, through the sclerotic portion of the conjunctiva and its subjacent cellular tissue, beginning at the margin of the cornea, and extending towards the edge of the orbit, in a direction as rays radiating from a centre, but avoiding immediately the transverse and perpendicular diameters of the globe; that the larger vessels, passing to the cornea, might not be injured. The plan being decided upon, I soon had opportunity of carrying it into effect." 75.

Several cases are related in illustration, but they do not require notice. They are very favourable to the plan, which we have, in two instances, tried ourselves with great benefit. Mr. Tyrrell concludes that the beneficial operation of the proceeding is only interfered with by two extremes—great excess of arterial action with fever; or extreme feebleness of vascular power.

"It is only, therefore, in cases in which such extremes exist, that I now deem it requisite to adopt other than simple and ordinary treatment generally, such as regulating the secretions, and allowing a diet adapted to the power and age of the patient, excepting when the chemosis is unusually firm or fibrinous, when I give mercurials to check such deposit.

When the force of the circulation is greater than natural, with or without febrile excitement, I abstract blood, but merely to such an extent, as to reduce the pulse a little below the ordinary standard. If the general power is much below what is proper, I immediately begin to promote its restoration, by a good and nutritious diet, with some stimulus, to which the patient has been accustomed, and frequently give also some tonic medicine. Locally, after the division of the chemosed part, I direct some simple fomentation to be used frequently, for twenty-four hours, and in case of pain continuing or returning, I direct leeches to be applied freely to the palpebræ, in number according to the degree of local action; after the pain has subsided, I recommend the fomentation to be made astringent, by the addition of some alum, and, as soon as the discharge acquires a whitish character, I discontinue the simple ointment, (at first employed to prevent the agglutination of the cilia,) and use, instead, a weak stimulating application, such as the diluted ointment of the nitrate of mercury, or the diluted ointment of the nitric oxide of mercury; and I further direct the strength of the lotion or fomentation, and of the ointment, to be gradually increased, or I make a change to some other astringent or stimulant, (which I generally find most advantageous,) till I perceive that all parts of the conjunctiva have recovered their healthy aspect.

When I have made the division of the chemosed membrane freely, I have not had occasion to repeat the operation; but when it has not been effectually done at first, in consequence of the excessive tumefaction of the eyelids, or from the resistance of the patient, I have had to repeat it; this has happened two or three times." 94.

When the entire cornea is opaque and dull, with complete chemosis, there is no prospect of saving vision. Yet the plan already recommended may be pursued, as most calculated to relieve pain, and to preserve what is preservable. It sometimes however happens, that only a portion of the cornea suffers, when the violence of the disease abates. The palpebræ lose their tension, their shining and florid color, and become of a purple hue, dull and somewhat corrugated; the conjunctiva becomes of a pale pink color, and flaccid; the secretion thin and whitish, although still often mixed with red particles of blood. All this should be noted, for, now a rough examination may destroy the eye. A sudden and careless pressure on the globe may force out the humours. The examination of an eye in this condition should, therefore, be conducted on a most cautious and delicate plan, so as to expose the cornea, with the least possible force; to obtain a proper view of the cornea some degree of force is requisite, in consequence of the swollen state of the palpebræ, but it is easy to apply the requisite force so gently and gradually as not to incur risk.

Another evil, observes our author, results from over-depletion—supposing the cornea to have suffered from a partial mortification, and the mortified part to have separated or sloughed off, no healing or healthy action ensues, the local power being inadequate to its institution; a transparent depression then denotes for a time the position and extent of the loss of substance, but unless some general or local means, or both, be resorted to, to excite a proper action, the exposed surface of the depression assumes a dull light brown, or dirty-looking aspect, becoming opake; and this appearance extends also in a small extent around the edges of the depression; after a little while this dirty-looking layer separates, and exposes again a transparent clear surface; but again, under exposure and deficient power, a second brownish opake slough forms and separates, and so on, until that which had at first been of trifling extent, becomes by degrees most formidable, and perhaps destructive of vision.

Local and general means should be resorted to to check this. The first may consist in the application by injection of a solution of nitrate of silver—one, two, three, four, or more grains, to an ounce of distilled water,—beginning with the weak solution and gradually augmenting the strength. The general treatment must be directed gently to support the patient, by diet, cautious stimulants, or tonics.

Mr. Tyrrell recommends, when the disease breaks out and prevails, as it too often has done, in any public institution, certain precautionary measures which we quote.

" First,—I should advise, therefore, that when the purulent ophthalmia attacks several individuals in the same community, they should be immediately separated from the general mass, and sent to some distant place, which would offer a decided change; but in which the soil should be dry, and free from exposure to the east wind.

Secondly,—that those who had been previously in close association with the infected parties, should be also separated from the general mass. I mean, particularly such as had slept with any of those in whom the complaint had appeared, or who had been in close contact with them, either for purposes of study or recreation. These parties might be put into a separate building, but should be kept from close intercourse, until sufficient time has elapsed, to determine whether they be infected or not; and they should be carefully examined at least twice in the day, that the earliest symptom of the disease might be detected, so that those in whom the disease appears, might also be immediately separated from the others.

Thirdly,—that the clothing, bedding, &c. of the infected persons, should be submitted to the ordinary modes of cleansing, and immediately afterwards exposed to a high temperature of dry heat, which appears to have a considerable influence in destroying any property of infection in such articles: and this plan might, perhaps, be advantageously adopted, with regard to the bedding and clothing of the uninfected, previously to their separation, individually, or altogether, from the residence in which the disease had appeared.

Then, fourthly,—that each and every one of those liable to the contagion, should also be carefully examined twice every day, in order to detect any disease, and to separate any infected parties.

Fifthly,—that every attention should be given to the cleanliness of every individual; that the diet and clothing should be such as would be likely to maintain a good condition of strength and temperature; and that the assemblage of many together in the same apartment, for more than a few minutes at a time, should be prevented.

Lastly,--that the apartments and buildings generally, in which those infected had resided, should be well cleansed and fumigated." 100.

*Purulent Ophthalmia in the Infant.*—Speaking of the treatment of this affection by the alum lotion, Mr. Tyrrell prefers to have it used just warm: and, at the time of its application, the upper eyelid should be gently raised by its integument, so as to allow some of the lotion to pass to the surface of the conjunctiva. This he thinks much better than the use of the syringe, which he is confident frequently does harm, for it is scarcely possible to use it without inflicting violence on the conjunctiva; and the inexperienced or incautious operator with it, runs great risk of getting the disease from some of the solution, with matter, getting into his own eyes.

When the *second stage*, that of chemosis, sets in, this is always indicated, says our author, by change in the palpebræ, which swell, and assume a red, tense, and shining character,—the extent of these symptoms being usually in proportion to the extent of the chemosis: this tumefaction of the eyelids is frequently such, as to prevent the surgeon from obtaining a view of the cornea, even by the exercise of great violence; it is therefore most satisfactory, that we can determine the condition of the disease by the aspect of the palpebræ, though we cannot at once form our prognosis, as when we can get a fair inspection of the cornea. So long as the eyelids are swollen and tense, and present a shining and florid surface, the cornea is generally safe, although it may be upon the verge of destruction; for no sooner is its vitality lost, in part, or in toto, than the tension of the palpebræ diminishes, the color becomes purplish, and the surfaces lose the shining or erysipelatous appearance; the discharge also becomes thinner and whiter; whilst under the circumstances first described, the secretion is thick and yellow. If the cornea can be exposed at the time that the palpebræ are swollen, and of brilliant color, it will rarely be found to be in any great danger, as the chemosis is very rarely then complete; on the other hand, when the cornea cannot be seen, in consequence of the swollen state of the eyelids, it is probable that the chemosis is great, perhaps complete, but it is not necessarily so.

As the surgeon cannot expose the cornea, or ocular conjunctiva, he cannot, of course, divide the chemosis. " He may, however," says Mr. T. " soon reduce the palpebræ, so as to obtain a view of the cornea; and, if necessary, he might make the division of the chemosed conjunctiva: this can be accomplished, by applying a leech or two to the surface of the eyelid, according to the age and strength of the little patient; after the leech has filled itself, and fallen off, bleeding from the bite should be encouraged, by the application of warmth and moisture, until the surface becomes nearly colourless, and somewhat flaccid: in children of two or three days old, a single leech will sometimes suck as much blood as will produce this effect, and in most cases it is necessary to arrest the bleeding after a short time, for the infant will not bear the loss of much red blood: by this means, the tension of the part being reduced, the cornea may be exposed with much less force, and, unless a great state of prostration should forbid it, the chemosis might be then divided, as in the adult."

Mr. Tyrrell has not yet tried the operation in the infant. If the disease proves destructive to vision, it generally is so from neglect. Out of a large number of cases in which the tumefaction of the eyelids has been excessive, (appearing almost bursting,) but in which the color has been florid and the surface shining, he hardly recollects a single instance of even partial slough of the cornea ensuing under the influence of local bleeding, to such an extent as to nearly destroy the color of the palpebræ; which usually also produces some general prostration.

After the abstraction of blood, the weak solution of alum should be used frequently tepid, and increased in strength, if necessary. The cilia and canthi should be lightly touched with a camel's hair brush dipped in some simple ointment—and, if the case proves obstinate, a weak solution of the nitrate of silver may be resorted to.

Should the cornea, on inspection, be found more or less hazy, and threatened with mortification, Mr. T. would advise division of the chemosis, four incisions being probably enough.

### Chronic Inflammation of the Conjunctiva following Purulent and Catarrhal Ophthalmia.

Mr. Tyrrell describes, under this head, the complaint commonly known as " granular lid." He looks on that complaint as the result of the ophthalmiæ mentioned, and believes that he has shewn that these diseases commence in the palpebral division of the conjunctiva, and from thence extend to the ocular portion. They disappear in the contrary order; leaving, first, the occular part of the membrane, or that in which they appear last, and linger in the palpebral portion of the tunic, or that in which they first appeared; and, in this division of the membrane, the morbid action may remain in so trifling a degree as to escape the observation of the careless practitioner, who may be satisfied with the perfect restoration of vision, independently of slight occasional interruption from a collection of superabundant secretion. In order to prevent the occurrence of the chronic affection, the palpebral conjunctiva should be carefully examined, when the acute disease appears to have been completely subdued; and if the membrane of the eyelid has not perfectly recovered its natural aspect, the remedies should be continued until all morbid appearance be subdued. The examination should extend to the conjunctiva of both eyelids.

He has, he assures us, repeatedly observed, that according to the severity of the acute stage, and in proportion to the extent of exhaustion created by the treatment employed, has been the risk and rapidity in the development of the chronic form.

We see nothing particular in Mr. Tyrrell's observations on this complaint. But his treatment of it when the stage of "granulated lid" has set in, that is, when the villi of the palpebral conjunctiva have decidedly enlarged, should be quoted. He first sets forth Mr. Saunders' plan of removing the villi with the knife, and comments on its inefficiency. He then relates a case illustrative of his method.—"I made frequent examinations of the diseased membrane; and, whenever I found it to be of a deep red colour and turgid, I directed a leech to be applied to the outer surface; but when it presented a lighter colour and was softer to the touch, I had applied immediately to it a more powerful astringent than I had previously used. That which I applied most frequently, and from which the most good resulted, was the *solution of the diacetate of lead* undiluted. It was smeared upon the diseased membrane by means of a camel's hair brush, the part being exposed by eversion of the eyelid, and cleansed from secretion by a piece of dry lint. Thus the astringent came in immediate contact with the morbid projections. I also occasionally applied the sulphate of copper, in substance, to the granular surface under similar circumstances; but it was used lightly, and not allowed to rest long enough in contact with the surface to produce an escharotic effect."

The plan, he informs us, answers, but he has occasionally modified it a little, having now and then incised the tumid membrane very lightly, by transverse incisions, extending the whole length of the tarsus; not so deep however as to injure the tarsus, but only to open the vessels of the diseased conjunctiva of the eyelid. He has usually made three or four parallel incisions in each superior eyelid; and afterwards has encouraged bleeding from the part, by the application of a sponge moistened with hot water; and then as soon as the vessels have been pretty well emptied, he has applied a powerful astringent.

In some few cases change of air has been requisite. When the granulations are large and flabby, the cure is generally slow. General treatment calculated to invigorate—the use of the undiluted solution of the diacetate of lead—and when the granulations are small, hard, and pale, the light employment of the sulphate of copper, are the measures that he recommends, and he adds repeated blisters behind the ears.

### Scrofulous Ophthalmia.

Mr. Tyrrell thinks, and most practical surgeons will agree with him, that there is no particular form of ophthalmia which, *par excellence*, can be called the scrofulous. The strumous constitution can modify them all, though the simple and pustular or phlyctenular forms are the most affected by it. The prominent feature is extreme intolerance of light.

On the subject of treatment Mr. Tyrrell has not much to say. General means and remedies are indispensable, of course. As local means, Mr. T. rarely employs any others than counter-irritation, by means of small blisters placed behind the ears, or above the eyebrows; never, however, continuing the irritation, by promoting discharge from the blistered surfaces by stimulating applications, but preferring the repetition of the blister. He generally uses simple warm water, or a decoction of poppy-heads or chamomile flowers, with, now and then, a few leeches to the palpebræ, when the conjunctival vessels are numerously and very fully distended with red blood.

"Many surgeons employ more powerful local agents in the treatment of this disease than those I have recommended; but, a very extended and impartial trial of such means, has induced me, by degrees, more and more to lay them aside, and adopt the simple plan which I have detailed. I have seen obstinate cases much benefitted, for a time, by the use of some severe counter-irritant, as the tartar-emetic ointment, issues, setons, &c.; but I have seen much more evil than good result from these means; and most sincerely believe that the disease is to be more effectually and permanently removed by the plan I have proposed. To astringent and stimulating applications to the organs themselves, I have very strong objections; arising from the observance of the frequent and serious mischief they produce.—I mean the common astringent and stimulating lotions, ointments, or drops. If they are employed at all, it should be when any general error of system, or important functional derangement has been removed; and I believe, that the advantage frequently obtained by their casual use at such a period, has led to a very exaggerated idea of their efficacy." 162.

We must say that, so far as we have seen, we are quite disposed to agree with Mr. Tyrrell.

### CHRONIC SCROFULOUS OPHTHALMIA.

The characteristics of this form of ophthalmia are the following:—

"In the most simple cases of chronic disease, the only differences which can be observed, exist in the number and colour of the vessels carrying red blood, which become less numerous, more tortuous, and of a darker or purplish hue, as the chronic form is assumed; and if there be discoloration of the palpebræ, such discoloration is also of a dark character. Otherwise, whatever may be the nature of the affection in its origin, whether simple, pustular, or catarrhal, as it affects the scrofulous person,—under continuance, it usually implicates nearly the whole of the ocular portion of the conjunctiva, as well as the palpebral. In the former, the vessels become large and tortuous, and of a dark red or purplish hue; and many pass from the sclerotic portion of the membrane to its corneal division: and whenever this occurs, the latter becomes thickened and loses its transparency, especially in those parts in which the vessels are rendered apparent, from being distended by red blood. In some cases, but few of these vessels are to be perceived, whilst, in others, they are exceedingly numerous; but the nebulous condition of the membrane is usually greater in proportion to the number of these vessels. This state of cornea is denominated *vascular and nebulous,* similar to that which results from the irritation of the granular eyelid as a consequence of chronic purulent or catarrhal inflammation; but there is a very marked difference in the mode of distribution of the vessels carrying red blood to the cornea. In the case of granular eyelid, they are rarely found encroaching upon the cornea in any direction, except from the upper part, as I have previously described; but in the scrofulous disease, they will be generally perceived passing over the sclerotic to the surface of the cornea, at nearly all points of the circumference of the latter, though the larger vessels usually take their courses from the directions of the recti muscles. Now and then also, we have a granular state of the eyelids. This, however, but rarely happens, unless the disease in its origin has been of a catarrhal or purulent kind." 165.

Indolent ulceration of the corneal part of the conjunctiva is common.

The subjects of this disorder are generally pallid, with a feeble circulation.

The treatment must, naturally, be directed towards the improvement of the general health. When the functions are improved, slight local stimulants, such as the vinum opii, or a weak solution of the bichloride of mercury, may be dropped into the eye once in twenty-four, or forty-eight hours. If the application creates a severe and continued pain, it rarely effects good, and should be laid aside; but if the smarting it produces does not exceed a few minutes in duration, leaving the eye tranquil, it may be continued usually with the best effect. Counter-irritation, in a very moderate degree, by small blisters applied behind the ears, may be resorted to when there is much intolerance of light, excepting when the powers of the patient are exceedingly depressed.

The more severe cases, in which the corneal conjunctiva is affected are exceedingly difficult to manage. The tendency to relapse is most troublesome. Every possible general precaution should be taken, and the general treatment should be such as to sustain without stimulating. Mr. Tyrrell speaks highly of Battley's solution of the yellow bark.

Mr. T. believes that warm water is the best local application, though stimulants are sometimes advantageous. Mr. T. speaks favourably of counter-irritation, but slightingly of setons and issues. He is confident that these are often prejudicial in children and in weakly persons. He relates two cases in support of this opinion.

### Exanthematous Ophthalmia.

This is—inflammation of the conjunctiva, with erysipelatous affection of the palpebræ. A rare disease, allied to the catarrhal.

The disease presents the following characters:—The eyelids are somewhat swollen, red, and shining, particularly at the margins; but the color is much lighter and the tension less, than is common in erysipelas; the cilia are partly loaded by a light yellow viscid matter, and some similar morbid secretion may also be usually found at the inner canthus: the palpebral conjunctiva is red, tumid, and villous; and the ocular part of the membrane is of a dirty yellowish red color, and elevated around the cornea by a deposit of serum into the subjacent cellular membrane, (*serous chemosis;*) this deposit is greatest at the lower part of the circumference of the cornea, and least at the upper part, from the gravitation of the fluid in the cells; it is also often irregular, causing protrusion of the membrane, at several points, giving the appearance of vesicles.

The patients who present this form of ophthalmia are generally weak, with disturbance of the digestive organs. They are generally, too, advanced in life and of irregular habits.

A few smart purges, strict diet, and the local use of a slightly astringent lotion and ointment will usually soon effect a cure. If there is much gastric uneasiness, an emetic proves of service.

The forms of ophthalmia, most common in the fevers which are attended by cutaneous eruption, are, the pustular and catarrhal, or muco-purulent: the latter commences with symptoms, so similar to those which usher in the purulent disease, that the medical man should be on his guard. If then, the conjunctival affection begin with much pain and heat, and there be a rapid change in the palpebral part of the membrane, so that it become thickened, villous, and of a deep carmine color, and at the same time the secretion present a yellow tinge, there is every reason to dread the development of the acute purulent inflammation; and it will most probably take place, unless means be pursued to check and subdue the first stage.

"Whilst in the first stage, the disease may usually be subdued by local bleeding, with leeches, and the subsequent use of warmth and moisture, with some slight astringent, as alum; the means appropriate to the cure of the febrile disease otherwise, as purgatives, abstinence, &c. will aid in subduing the local complaint; but should the attack commence with severe pain, and rapid tumefaction of the conjunctiva, with other symptoms of acute kind, and the circulation be full and incompressible, and the patient possessing good constitutional power, I can discover no good reason why the force of the circulation should not be lessened, by abstraction of blood from the arm; it would probably lessen also the violence of the febrile disease, and expedite recovery: the surgeon should, of course, be extremely cautious not to remove more blood, than would be requisite to reduce the force of the circulation a little below the ordinary standard, and not so much as to produce prostration. I have seen this plan adopted in the first stage of purulent ophthalmia, occurring with small-pox, and with the best effect, generally, as well as locally. The remedy is, however, a hazardous one, and should not be adopted, without a perfect conviction of the power of the patient being good, and the action of the heart and arteries being above par, not in frequency, but in force." 181.

We would recommend surgeons to be very cautious in employing general bleeding in the exanthemata. We would undoubtedly pause ere we resorted to it.

The local disease comes on at various periods of the febrile attack; sometimes quite in the commencement: occasionally during its height; and, sometimes, as the severity of the general disease is passing off. Mr. T. prefers general bleeding when the ophthalmia is developed in the early stage of the fever. When chemosis, &c. have supervened, Mr. T. would adopt nearly the same plan as if the fever were not present, though he would be more cautious in lowering the patient.

During, says our author, the continuance of, or subsequent to some of the exanthematous diseases, when the febrile action has been unusually severe, and has occasioned great prostration of the general power, inflammation of the conjunctiva sometimes arises, attended with ulceration of the cornea, by which this transparent texture usually suffers extensively; the progress of the ulcerative process being rapid. Mr. Tyrrell has seen the same thing in cases of typhus fever.

"I would recommend," says Mr. T. "that the treatment should be such, as is best calculated to promote and maintain a good state of the general power; as, a nutritious diet, with the moderate use of an accustomed stimulus, tonic medicines, with proper regulation of the secretions, and the use of mild astringents, or stimulating lotions to the eyes. I should not advise the application of any powerful stimulus locally, until the general power is much improved: for, during the state of great debility, a strong stimulus may excite a degree of action, which the part is not able to support; and more extensive sloughing may result. A solution of nitrate of silver, injected upon the surface of the ulcer or depression, may be of service, when the general power is considerably re-established, and no healthy local action appears to take place." 184.

We have completed a notice of the ophthalmiæ, and have extracted such facts or opinions as we think are calculated to be useful. In our next number, we shall take up the remaining portions of the work, and complete our account of it. We need scarcely add, that we consider it a very valuable addition to the existing books upon diseases of the eye, and we believe that it will prove eminently useful.

